# Metabolomics and Biomarkers in Retinal and Choroidal Vascular Diseases

**DOI:** 10.3390/metabo12090814

**Published:** 2022-08-30

**Authors:** Xiao-Wen Hou, Ying Wang, Chao-Fu Ke, Mei-Yan Li, Chen-Wei Pan

**Affiliations:** 1 School of Public Health, Medical College of Soochow University, Suzhou 215123, China; 2 Department of Ophthalmology, EYE & ENT Hospital of Fudan University, Shanghai 200031, China

**Keywords:** metabolomics, retinal neovascularization, diabetic retinopathy, retinopathy of prematurity, neovascular age-related macular degeneration

## Abstract

The retina is one of the most important structures in the eye, and the vascular health of the retina and choroid is critical to visual function. Metabolomics provides an analytical approach to endogenous small molecule metabolites in organisms, summarizes the results of “gene-environment interactions”, and is an ideal analytical tool to obtain “biomarkers” related to disease information. This study discusses the metabolic changes in neovascular diseases involving the retina and discusses the progress of the study from the perspective of metabolomics design and analysis. This study advocates a comparative strategy based on existing studies, which encompasses optimization of the performance of newly identified biomarkers and the consideration of the basis of existing studies, which facilitates quality control of newly discovered biomarkers and is recommended as an additional reference strategy for new biomarker discovery. Finally, by describing the metabolic mechanisms of retinal and choroidal neovascularization, based on the results of existing studies, this study provides potential opportunities to find new therapeutic approaches.

## 1. Introduction

Vasculogenesis and angiogenesis are two processes that are indispensable for the formation of vascular networks, and they both promote neovascularization in a specific microenvironment [1,2]. Neovascularization in the physiological state contributes to wound healing. Pathological neovascularization results when exogenous stimuli, such as hypoxia and ischemia, disrupt the regulation of the dynamic balance between endogenous pro-angiogenic and anti-angiogenic factors [3]. Abnormal neovascularization is associated with many diseases, including cancer, hypertension, and ocular neovascularization disease.

The retina is one of the most important structures in the eye, converting incident light into neural signals that can be received by the visual cortex of the brain, thus allowing us to receive information from the outside world. The high metabolic demands of the retina are met by a well-organized vascular system. The types of neovascularization affecting the retina include both retinal neovascularization and subchoroidal or choroidal neovascularization (CNV) [4]. Incomplete endothelial cells of neovascularization undergo spontaneous rupture, producing fundus hemorrhage, and exudation, leading to loss of central vision, visual distortion, and reduced contrast sensitivity in patients, eventually progressing to irreversible vision loss and leading to blindness. Neovascularization associated with retinal vascular disease is a comprehensive phenotype of a group of diseases, such as diabetic retinopathy (DR), retinopathy of prematurity (ROP) and neovascular age-related macular degeneration (nAMD), pathological myopia, and idiopathic CNV, and associated risk factors have been reported in different studies [5]. Frequently reported risk factors include age [6], hyperglycemia [7], diet [8], transmissibility, trauma, and inflammation [9]. Although considerable progress has been made in understanding the risk and genetic architecture of neovascularization, the exact etiology is still not fully understood.

Metabolomics offers the opportunity to analyze the endogenous small molecule metabolites of an organism, conveying “signals from genetic structure and environment” and providing “functional readouts of physiological states”. Khan et al. recommend it as a promising histological technique for disease investigation [10]. Metabolites can serve as dynamic indicators of the interaction of genes, proteins and the external environment in physiological and pathological states, and identifying metabolites with strong quantitative differences between phenotypic classes (e.g., normal and disease samples) may be an ideal way to find disease-related biomarkers in the context of a hypothesis generation-driven research approach [11]. The ideal biomarker should have balanced sensitivity and specificity, be easily accessible, be both non-invasive and cost effective, and provide the opportunity for early detection of disease, and it should exist as a quantitative indicator of disease status [12]. Abnormal states, such as inflammation, alter the permeability of the blood-ocular barrier, making it possible to study neovascular diseases through plasma and serum metabolomics [13]. However, limited relevant studies exist and the design and analysis still need to be rationalized and standardized, which also results in differences in biomarkers identified by different studies and the need to find criteria and methods to enhance biomarker performance. This review summarizes the existing study designs and results, and, finally, attempts to use the existing findings to explain disease mechanisms and facilitate the clinical translation of metabolomics findings.

## 2. Application of Metabolomics in Retinal and Choroidal Neovascularization Studies

The eye is a relatively independent organ, and the blood-ocular barrier system regulates intraocular fluid content and maintains a relatively stable state of metabolic environment in the general circulation of the body [14]. The blood-retinal barrier is a physiological barrier to metabolic exchange in the retina, divided into internal and external barriers. Retinal capillary endothelial cells limit the efflux of plasma and its components into the interstitial space of the retina, forming an internal blood-retinal barrier. Retinal pigment epithelial cells located between the choroid and the outer segments of photoreceptor cells form the outer blood-retinal barrier, which separates retinal tissue fluid from choroidal tissue fluid and has selective permeability and active transport functions. The choroidal vessels accommodate up to 85% of the blood volume in the eye, allowing plasma to bathe the retinal pigment epithelium (RPE) and meet the high metabolic demands of the photoreceptors [15].

When exogenous stimuli cause pathological neovascularization, it disrupts the barrier system and the diffusion barrier between the retinal extracellular fluid and the adjacent vitreous disappears, disrupting the local environmental homeostasis of the aqueous humor (AH) and vitreous, which also offers the possibility to study neovascular disease through ocular tissue and blood [13]. Neovascularization is a common pathological manifestation of a group of vascular diseases involving the retina, of which CNV is mainly a proliferation of neovascularization in the choroidal capillary layer that breaks through and grows across Bruch’s membrane toward the retina and enters between Bruch’s membrane and RPE or between the RPE layer and the retinal neurosensory layer, ultimately affecting vision, of which nAMD is the main representative [16]. Neovascularization of the retinal capillary network is represented by DR and ROP [17]. There is great etiological variation among these eye diseases, but they exhibit the same neovascularization phenomenon, which may arise as a cumulative result of gene-environment interactions. Therefore, metabolomics studies may contribute to the understanding of disease mechanisms. This section summarizes studies on the use of metabolomics platforms to reveal metabolic changes in several classes of diseases presenting with retinal and choroidal neovascularization changes, including AMD, PDR, and ROP. Metabolites that undergo changes in pathological states are summarized and the mechanisms of disease are further elucidated on the basis of pathway analysis. We emphasize the importance of technical and biological validation and clinical utility assessment, advocating a comparative results strategy based on existing studies, which covers both performance optimization of newly identified biomarkers and consideration based on the existing research base as additional optimized reference standards for the discovery of desirable biomarkers.

### 2.1. Metabolomics to Identify Metabolic Changes in Retinal and Choroidal Neovascular Disease

**Figure 1:**
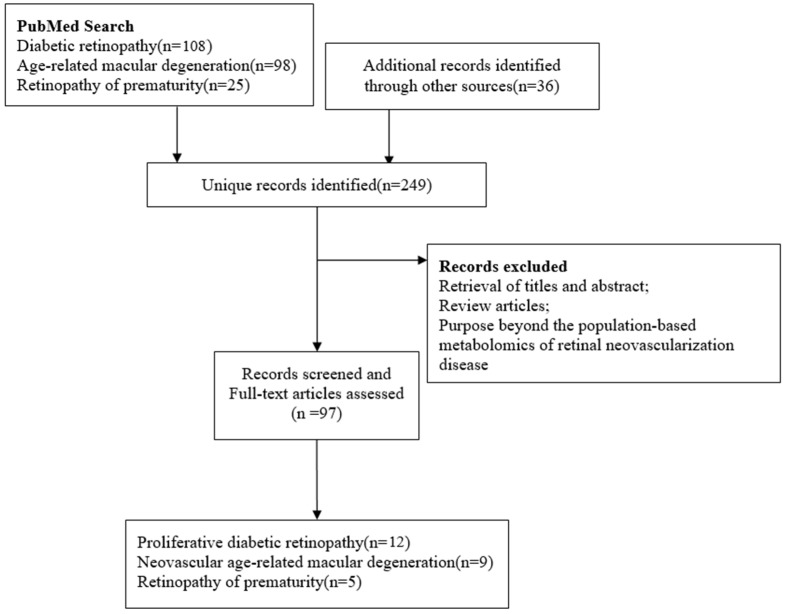
Flow diagram of literature search and study selection for metabolite markers of retinal neovascularization disease.

Metabolomics studies have reported several ocular diseases associated with neovascularization, including AMD, DR, and ROP, and we used these three common eye diseases as examples to develop a review of metabolic changes. We conducted a literature search using the PubMed database. Articles were identified by searching for titles using the following search terms: (“metabolomics” or “metabonomics” or “metabolome” or “metabolic profiling”) AND “neovascularization-related disease*”. Additional articles were identified by searching the reference lists of the included studies. We included metabolomics studies based on mass spectrometry (MS) or nuclear magnetic resonance (NMR), focusing on the generalization of population-based studies presenting with neovascularization pathological states and looking for differential metabolites, excluding drug evaluation reports, review articles, and abstracts without full text, resulting in the inclusion of twenty-six studies, including five studies of ROP, nine studies of nAMD, and twelve studies of DR. A simplified flow diagram of study selection is shown in Figure 1. Important details about the study design and methods were extracted from selected articles and summarized (Table 1).

All studies were observational, and all 25 studies were case-control studies, except for a longitudinal multi-time point observational study of serum in preterm infants established by Nilsson et al. [18]. Twelve studies (46.15%) were conducted in China [19,20,21,22,23,24,25,26,27,28,29,30], four (16%) in the United States [31,32,33,34], two (8%) in Japan [35,36], and the remaining studies were conducted in Spain, Korea Switzerland, and elsewhere. [18,37,38,39,40,41,42,43] The studies were published between 2009 and 2022, with sample sizes ranging from 40 to 431, and non-equilibrium rates (number of cases in the majority category/number of cases in the minority category) between the case and control groups of the sample ranging from 1 to 3.66. Factors such as age, gender, race and lifestyle affect metabolic processes, with the ROP study recruiting newborns as study participants and both the nAMD study and the DR study recruiting middle-aged and older participants. The study by Mitchell et al. had the lowest proportion of males in the nAMD group (35%) [32] and the study by Tomita et al. study had the proliferative DR group with the highest proportion (77.1%) [36]. Smoking was present in some studies, especially involving elderly populations. Reasonable control of the influence of biological and experimental confounding factors is also important to ensure the reliability of the study results. Some studies reported measures such as collection of biological samples after eating, gender and age matching. Finally, the selection of study controls affects the interpretation of the study results, but due to ethical factors and sample availability, biological rock samples of controls are often obtained from other diseases or other procedures in our department, and not healthy controls, such as those in the study by Han et al., whose study control samples were obtained from cataract patients without AMD [22], Zhu, Sumarriva et al. used plasma from NPDR subjects [26,33], and Wang et al. obtained vitreous samples from MH patients and AH from cataract patients [21]. Regarding analytical platforms, 24 articles reported mass spectrometry-based metabolomics studies, including liquid chromatography-mass spectrometry (LC-MS; *n* = 22), and gas chromatography-mass spectrometry (GC-MS; *n* = 2), with different detection platforms covering different ranges of metabolic pouches, which also contributed to the differences in detection across studies.

**Figure 2:**
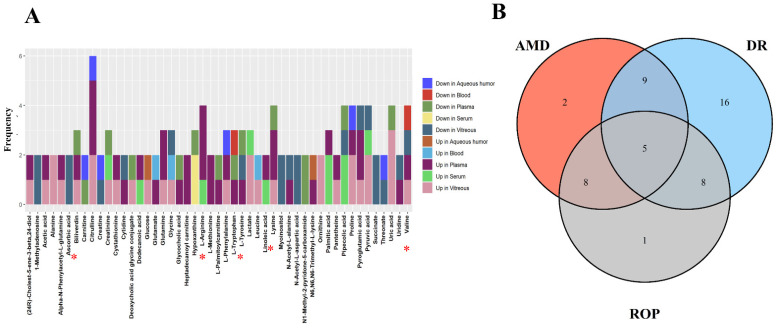
(**A**) horizontal coordinates indicate the metabolites identified repeatedly in the study, vertical coordinates represent the frequency of repeated identification, colors represent the direction of metabolite variation across biological samples, and red asterisks mark the differential metabolites found in all three diseases. (**B**) represents the Venn diagram of the metabolites identified repeatedly in the different diseases. * Red asterisks indicate the five differential metabolites identified in three diseases.

The included studies generally utilized a combination of univariate and multivariate analyses to screen for between-group differential metabolites, with 11 of the total studies utilizing supervised multivariate analysis methods, such as partial least squares/orthogonal partial least squares discriminant analysis, in combination with univariate analysis methods, such as *t*/U test/univariate ANOVA/Wilcoxon rank sum test [19,20,21,22,25,26,27,28,29,30], with 4 of the 10 studies additionally adding fold change as a screening criteria [19,28,29] and one adding univariate area under curve value as an additional screening criterion [26]. It is worth noting that further screening criteria for difference variables often differed between studies, with criteria set at variable importance in projection (VIP) values greater than 1 or 2 and *p* values less than 0.05 or 0.01 for univariate analysis, or a Benjamini-Hochberg correction to control for false discovery rates. VIP values were used to screen metabolites by measuring the impact and explanatory power of metabolites on the increase in categorical discrimination of each group of samples in [44], while *p*-values were the probability of occurrence of more extreme results than those observed in the obtained samples in [45], and ploidy change methods, i.e., calculating the fold of difference in expression of a metabolite between two groups based on the relative quantitative or absolute quantitative results of the metabolite. Different analytical methods observe data from different perspectives, and different methods can be used in combination depending on the actual study purpose. In the included study, we counted significant metabolites and their trends (Figure 2). There were 38 metabolites reported repeatedly in different biological samples, 12 of which were reported more than, or equal to, 3 times. Among them, Citrulline appeared most frequently (*n* = 6), followed by Arginine, Lysine, Proline and Pyruvic acid.

### 2.2. Biomarker Interpretation and Optimal Selection

Some of the reasons that prevent biomarkers identified by experimental studies to be used in clinical applications are the difficulty of explaining changes in complex disease mechanisms by individual biomarkers [46] and the difficulty of validating the results in other validation sets due to the strict limitations of sample screening [47]. Metabolism is a dynamic process, and metabolic data respond to changes in endogenous and exogenous metabolites at a particular point in time [48]. Within an organism, metabolites coordinate with each other to form a complete network of biological functions, and it is difficult to explain complex disease mechanisms with a single metabolite. Pathway analysis is a way to interpret and provide more biofunctional information about biomarkers by mapping metabolites that are changed by abnormal disease states onto an interactive network constructed with a priori knowledge, by mapping individual changed biomarkers into a coherent metabolic network [49]. In this review, biochemical interpretation of all altered metabolites was performed using MetaboAnalyst 5.0 (http://www.metaboanalyst.ca/, accessed on 1 August 2020), a software that allows analysis of the impact of particular compounds on biochemical pathways. Pathway analysis was derived from integrating differential metabolites based on the Kyoto Encyclopedia of Genes and Genomes and Human Metabolome Database [50]. We performed separate pathway analysis of the differential metabolites identified in our study in different biological sample sources, and the results of the study showed that 8, 4, 4 and 10 statistically significant metabolic pathways (*p* < 0.05) were enriched in plasma, serum, AH and vitreous humor, respectively. Table 2 shows the results of pathway analysis in each biological sample (FDR < 0.05) and matching differential metabolites in each pathway. Arginine biosynthesis, Aminoacyl-tRNA biosynthesis and Glyoxylate and dicarboxylate metabolism pathways were repeatedly enriched in different biological samples. When pathway analysis was performed separately, according to disease type, the following four metabolic pathways were repeatedly enriched (*p* < 0.05): Phenylalanine, tyrosine and tryptophan biosynthesis; Aminoacyl-tRNA biosynthesis; Linoleic acid metabolism; Lysine degradation. Among them, the Aminoacyl-tRNA biosynthesis pathway was meaningfully enriched in the analysis of different biological samples and disease species, respectively. It is worth noting that the existing databases, such as KEGG, include the results of existing studies, and more detailed information on metabolic pathways needs to be further improved. Therefore, the *p*-values and Q-values of the pathway analysis are only a reference for the study results, and those pathways that are not significant are also worth interpreting.

The focus of this study was to review metabolomics studies of the pathological phenomenon of retinal and choroidal neovascularization, and, therefore, included differential metabolites among subjects with different disease sources, biological samples, ages, and ethnicities, and screened for repeatedly identified differential metabolites. Biomarkers should have the ability to help identify diseases, and nine studies in this review evaluated the intergroup discriminatory performance of identified potential biomarkers [51], as shown in Table 3. Only the study by Mitchell et al. did an internal validation (test set) for identifying a panel of metabolite markers (159 differential features), obtaining an AUC value of 0.83 AUC value [32]. Ideal biomarkers need to be evaluated and compared in multiple ways, and repeatedly identified differential metabolites may become biological representations of disease characteristics. We validated the extent to which repeated identification of differential metabolites contributed to disease classification using the raw metabolic data mentioned in the four studies included in the review, including the study by Han et al. that included both anionic and cationic patterns and the study by Wang et al. that covered both vitreous and AH biological samples [21,22,37,42]. We randomly divided the case and control groups into training and validation sets containing 70% and 30% of these samples [52], respectively, and used repeated identification of differential metabolites as metabolic biomarker panels to construct multiple machine learning models for repeated identification of differential metabolites, including logistic regression, random forest, support vector machine (SVM) and XGBoost, and performed model evaluation. The results are shown in Table 4. The results show that repeated identification of differential metabolites had good classification performance in different studies and models. In addition, the study suggested that among the included metabolomics studies, random forests obtained better performance in small sample studies and n/p large differences. XGBoost showed better performance in unbalanced sample data and SVM and integration methods were more stable in studies with relatively small sample sizes and numbers of features. The results provide an additional reference for researchers to compare with the gold standard when identifying biomarkers in the future, in addition to duplicate identification markers in studies, and it covers both performance optimization of newly identified biomarkers and considerations based on the existing research base.

Random Forest (RF) is a statistical learning theory, which uses a bootstrap resampling method to draw multiple samples from the original sample, model decision trees for each sample, and then combines the predictions of multiple decision trees to arrive at the final prediction by voting. Support vector machines (SVM) are based on a model that defines “the classifier with the largest interval on the feature space”. The extreme gradient boosting (XGBoost) algorithm is based on the idea of boosting, which is an optimization improvement of the gradient boosting decision tree, and is a collection of many CART regression tree models to form a strong classifier. AUC = area under curve.

## 3. Metabolomics for the Interpretation and Treatment of Retinal and Choroidal Neovascularization

A well-functioning vascular system is necessary to meet the high-energy metabolic demands of the eye and the function of retinal neurons, and pathological states of the vascular system (neovascularization) are accompanied by a range of metabolic changes and occur in a range of ocular diseases. Metabolomics is being used to unravel disease-associated metabolic disorders and also offers the opportunity to find new therapeutic targets, employing angiogenic phenotypes that may reprogram the metabolism of disease [53].

### 3.1. Amino Acid Metabolism and Neovascularization

A growing number of studies have shown that amino acid metabolism affects functions such as vascular tone, redox homeostasis, and immunity and inflammation, and amino acids with different characteristics have different functions [54,55]. In this study, many amino acid metabolism-related pathways were found to be separately enriched in different biological samples and diseases. Glutamine was identified as a differential metabolite in three studies and is the most abundant free amino acid in plasma [21,25,41], participating in the tricarboxylic acid cycle, providing precursors for gluconeogenesis and converting to glutamate and ammonia by the action of glutaminase. Glutamate is a retinal excitatory neurotransmitter, and high concentrations of glutamate overstimulate ionotropic glutamate receptors (N-methyl-D-aspartate receptors), leading to overload of intracellular calcium ions and ultimately cell death [56]. Glutamate is often involved in maintaining important functions of the retina as a downstream product of, or related to, other amino acid metabolic pathways. Arginine induces the gene expression of insulin-like growth factor 1 (IGF-1) [57], and IGF-1 is involved in vascular neogenesis by enhancing the vascular endothelial-derived growth factor (VEGF) signaling pathway [58], which was observed to be upregulated in four studies [24,27,41,43]. The metabolism of arginine is regulated by nitric oxide synthase (NOS) and arginase, and its metabolites could mediate a variety of angiogenic reactions. L-arginine is catalyzed by NOS to produce nitric oxide (NO), one kind of multifunctional messenger molecule. Numerous studies have shown NOS and NOS-produced NO involvement in promoting VEGF-induced vascular permeability and angiogenesis through the regulation of endothelial cell function [59,60,61]. Palmer et al. found that arginine deficiency in endothelial cell cultures was associated with eNOS dysfunction [62]. Arginase competes with NOS to metabolize L-arginine to produce urea and ornithine. Increased activity of arginase could decrease the availability of L-arginine for NOS, thus reducing bioavailability of NO production, increasing formation of reactive oxygen species, and ultimately resulting to endothelial cell dysfunction [63]. Proline is the downstream product of arginine metabolism and plays a key role in maintaining cell redox homeostasis. It was elucidated that L-proline could reduce the level of NO related marker P-asymmetric dimethylarginine and improve the redox state, thus increasing the bioavailability of NO [64]. Proline is converted to glutamate by further metabolism [65]. In addition, pathways related to branched chain amino acids (BCAAs) (valine, leucine and isoleucine biosynthesis) were found to be significantly enriched in tissue biological samples (AH and Vitreous). Of these, valine was identified in four studies [21,23,30,38], and, interestingly, valine levels showed an opposite direction of change in two DR studies, probably due to differences in study populations and analysis platforms, while leucine showed an increasing trend in both studies. Higher BCAAs level are associated with increased neurotoxic levels of glutamate in the retina [66]. BCAAs exert their function through activation of the mammalian rapamycin (mTOR) pathway, which regulates cell growth, proliferation, and survival [67]. MTOR plays a key role in upregulating the VEGF pathway, which leads to augmented expression of caspase-3 and promotes proliferation and migration of endothelial cells [68,69]. Complement factor H is a tyrosine sulfated protein and there is an association between its specific haplotype and an elevated risk of developing nAMD [70,71]. Complement factor H on the RPE and choroid regulates the formation of the local membrane attack complex, which is essential for the development of laser-induced CNV [72]. Inhibitors of amino acid-related metabolic pathways have important therapeutic value for diseases caused by pathological neovascularization. However, further studies are still needed.

### 3.2. Abnormalities in Neovascular Metabolism and Potential Therapeutic Opportunities

The active metabolism of the retina requires large amounts of energy and oxygen consumption. Vascular dysfunction may lead to oxidative stress and excessive production of reactive oxygen species, resulting in oxidative damage [73]. The process of neovascularization also requires significant oxygen consumption, which can further exacerbate retinal hypoxia. Hypoxia-inducible factor-1α (HIF-1α) is a specific mediator in adaptation to hypoxic environments and pathological responses [74]. HIF-1α deficiency in a nude mouse model significantly reduced the growth rate of malignant tumors and decreased neovascularization [75]. Under hypoxic conditions, HIF-1α enters the nucleus and binds to HIF-1β to form dimers, which finally bind to hypoxia response elements; the bound dimers promote angiogenesis by inducing upregulation of the expression of VEGF, erythropoietin, glucose transporter 1 and platelet-derived growth factor (PDGF) [76]. PDGF is a hypoxia-regulated gene product that, together with VEGF-A, promotes ocular neovascularization, and the combination of the two causes an additive effect [77]. PDGF inhibitor (Fovista; Ophthotech, New York, NY, USA) has been proposed in combination with anti-VEGF (ranibizumab) for the treatment of nAMD, and results from a phase 2b clinical trial showed significant improvement, compared to anti-vascular endothelial growth factor monotherapy [78]. However, to the disappointment of the clinical investigators, the next two phase 3 trials did not show the benefits of the combination therapy.

Oxidative stress mainly affects RPE cells [79]. Proline was identified in four studies and was reported to mediate metabolism between RPE cells and the retina, to act as an alternative energy source during stress or hypoxia, and to act as an antioxidant in maintaining redox homeostasis by controlling mitochondrial function [80]. Oxidative stress also disrupts the regulation of the complement system by RPE cells [81], which is involved in innate immune response and, once activated, forms membrane attack complexes [82]. Nozaki et al. showed that somatic induction of endochoroidal neovascularization increased the levels of C3a and C5a, and that C3a and C5a induced an increase in VEGF secretion by primary human RPE in vitro [83]. Knockout of C3 gene protected mice from choroidal neovascularization after laser treatment. Complement pathway-related therapeutic agents have been proposed, such as an inducer targeting C5 (ARC1905) used in clinical trials for the treatment of nAMD and geographic atrophy [84] and the therapeutic complement receptor 2-factor H used to inhibit angiogenesis and RPE injury in a mouse model of CNV [85].

Endothelial cells rely on glycolysis to obtain ATP, and upregulation of pyruvate (an intermediate product of glycolytic metabolism) was observed in both serum and vitreous metabolomics studies in [21,28,36] The end product of glycolysis, lactate, contributes largely to the angiogenic phenotype through inhibition of prolyl hydroxylase 2 and activation of HIF1α and nuclear factor-κB [86]. In this study, the Citrate cycle (TCA cycle) pathway was found to be enriched, and previous studies found that oral supplementation with TCA cycle metabolites helped maintain retinal function and protected photoreceptor cells and nerves on the inner retinal network in mice [87]. A recent study by Kanow et al. highlights the importance of maintaining a gradient of aerobic glycolytic activity between the retina and the RPE [88]. When the glycolytic activity of the RPE is abnormally high, less glucose is available for retinal consumption, which can also lead to photoreceptor death. This is evidenced by the upregulation of lactate observed in three studies. VEGF enhances the expression of the key glycolytic enzyme 6-phosphofructo-2-kinase/fructose-2,6-biphosphatase 3 (PFKFB3), increasing glycolysis to support the high ATP requirement required for angiogenesis [89,90]. Pharmacological blockade of PFKFB3 has been shown to effectively impair pathological angiogenesis and enhance the anti-angiogenic effects of anti-VEGF in CNV models [89]. Extracellular purine metabolites (ATP, ADP and adenosine) are important regulators of vascular homeostasis. It was shown that adenosine A2a receptor (ADORA2A) activation promotes transcriptional induction of glycolytic enzymes through the HIF-1α pathway, whereas knockdown of ADORA2A reduces hypoxia-induced glycolytic enzyme expression and glycolytic flux and inhibits endothelial cell proliferation and angiogenesis [91]. These findings provide directions for the development of therapeutic drugs targeting the glycolytic pathway for retinal neovascularization.

Pathway analysis revealed that Arachidonic acid metabolism, Biosynthesis of unsaturated fatty acids, Glycerolipid metabolism and Linoleic acid metabolism pathways were significantly enriched, suggesting that fatty acid metabolism may be involved in the mechanism of retinal and choroidal neovascularization. Retina is a tissue rich in polyunsaturated fatty acids (PUFA), which are metabolized mainly by cyclooxygenase, and lipoxygenase, and cytochrome P450 enzymes, 12-hydroxyeicosatetraenoic acid (12-HETE) and 20-hydroxyeicosatetraenoic acid (20-HETE), are among the metabolites [92,93,94]. It is thought that 12-HETE is an important regulator of neovascularization that regulates the expression of VEGF and PEDF. Arachidonic acid metabolism via cytochrome P450-4A/F produces 20-HETE, which has been shown to stimulate c-Src and epidermal growth factor receptor-mediated downstream signaling pathways, including MAPK and PI3K/Akt pathways, the eNOS uncoupling, and NOX/ROS system activation, thereby inducing the proliferation, migration, secretion, and angiogenesis of pro-angiogenic molecules (e.g., HIF-1α, VEGF) in endothelial progenitor cells. In vivo and in vitro studies have shown that eicosapentaenoic acid (EPA) inhibits the activation and expression of VEGF-specific tyrosine kinase receptors, while EPA supplementation significantly reduces the mRNA expression and protein levels of ICAM-1, MCP-1, VEGF and IL-6 [95,96,97], providing an opportunity to find new therapeutic targets. The number of studies is currently limited, and some studies of related diseases exist outside of the study to provide complementary findings [98]. Biomarkers and the search for disease-related metabolic pathways contribute to our understanding of disease mechanisms, but are not yet clinically translatable and require continuous research. In addition, metabolomics studies require strict quality control, from sample sources, inclusion criteria, analytical platforms, and data processing and analysis, which can all affect the results.

## 4. Conclusions

With the advances in histological technologies, more and more features of diseases at the molecular level of genes, proteins, metabolism, etc. have been obtained and have unveiled a new understanding of disease features. Retinal and choroidal neovascularization disease is a common feature of a group of diseases and is one of the serious pathologies that threaten visual function. Current metabolomics studies have identified metabolic changes in selected retinal and choroidal neovascular diseases, identified disease-related metabolic pathways, and provided new insights into understanding disease mechanisms. However, metabolomics study design is critical to the accuracy and reproducibility of findings, so this study summarized existing studies and advocates a comparative strategy, based on existing studies, as an additional guiding strategy to direct new biomarker discoveries. In addition, the study discusses existing metabolomics findings to promote understanding of disease mechanisms and potential therapeutic opportunities to provide additional therapeutic opportunities to the current treatment strategy based on anti-vascular endothelial factor therapy.

## Figures and Tables

**Table 1:** Characterization of metabolomics studies of retinal neovascularization events.

References	Comparison	Age	Male%	Source/Race	Biofluid	Technique Employed	Evaluation Standard
ROP							
Yang (2020)	ROP patients (*n* = 40)	GA (31.20 ± 4.62 weeks)	37.50	China	Blood	UPLC-MS/MS	OPLS-DA (VIP > 1) & *t*-tests (*p* < 0.05) & Wilcoxon (*p* < 0.05)
	controls (*n* = 41)	GA (30.96 ± 4.17 weeks)	73.17				
Nilsson (2021)	47 ROP cases			Sweden	Serum	LC-MS	Repeated measures data (Spline function)
	cord blood and at postnatal days 1, 7, 14, and 28, and at postmenstrual weeks 32, 36, and 40						
Zhou (2021)	ROP patients (*n* = 58)	29.09 ± 2.23 (weeks)	58.62	China	Plasma	HPLC-MS/MS	Mann-Whitney U test (*p* < 0.05)
	controls (*n* = 25)	31.29 ± 2.33 (weeks)	52.00				
Zhou (2020)	ROP patients (*n* = 38)	GA (29.28 ± 2.42)	55.26	China	Plasma	UHPLC-MS	OPLS-DA (VIP >1) and *t*-test (*p* < 0.05/0.05 < *p* < 0.1)
	controls (*n* = 24)	30.61 ± 2.75	56.52				
Ozcan (2020)	ROP patients (*n* = 26)	GA (28.5 ± 2.7)		Turkey	Plasma	LCMS/MS	Mann-Whitney-U test (*p* < 0.05)
	controls (*n* = 29)	31.52 ± 2.6					
AMD							
Mitchell (2018)	NVAMD patients (*n* = 100)	79.2	35.00	America	Plasma	LC-MS and LC-MS/MS	Nested feature selection
	controls (*n* =192)	71.9	36.00				
Luo (2017)	NVAMD patients (*n* = 20)	66.20 ± 11.51	55.00	China	Plasma	UPLC-QTOF MS	PLS-DA (VIP > 1) & *t*-test (*p* < 0.05 or 0.05 < *p* < 0.1)
	controls (*n* = 20)	64.70 ± 11.60	55.00				
Osborn (2013)	NVAMD patients (*n* = 26)	76.0 ± 5.7		Caucasian	Plasma	LC-FTMS	Multiple testing
	controls (*n* = 19)	76.4 ± 4.8					
Li (2016)	PCV (*n* = 21)	60.7 ± 9.4	62.00	China	Serum	UPLC-MS	OPLS (VIP > 1), *t*-test (*p* < 0.05)
	controls (*n* = 19)	64.8 ± 9.2	53.00				
Barca (2020)	NVAMD patients (*n* = 40)	81.1	39.00	France	Plasma	LC MS	*t*-test (*p* < 0.05)
	controls (*n* =40)	81.8	41.00				
Liu (2020)	AMD (*n* =88), PCV (*n* = 102), PM (*n* = 57)	69.84 ± 8.47 (AMD), 66.06 ± 11.42 (PCV), 55.32 ± 14.49 (PM)	66.28 (AMD), 71.57 (PCV), 28.07 (PM)	China	Serum	GC-TOF-MS	OPLS-DA (VIP > 1.0), *t*-test (*p* < 0.05), and FC > 1.2 or <0.8
	controls (*n* = 81)	65.83 ± 11.94	35.80				
Han (2020)	nAMD patients (*n* =26)	74.12	53.85	China	AH	UHPLC-MS/MS	OPLS-DA (VIP > 1) & one-way variance (*p* < 0.05)
	Cataract patients without AMD (*n* = 20)	69.6	65.00				
Deng (2021)	127 nAMD (CNV + PCV)	71.1 ± 8.4	61.00	China	Plasma	UHPLC-MS	PLS-DA (VIP ≥ 1), FC ≥ 2 and FC ≤ 0.5, *p* < 0.05
	controls (*n* = 50)	68.5 ± 9.0	61.00				
Lambert (2020)	nAMD (*n* = 72)		38.89	European	Serum	NMR	one-way ANOVA
	controls (*n* = 50)	74.96 (6.24)	48.00				
PDR							
Sumarriva (2019)	PDR patients (*n* = 34)	55.7 ± 10.9	65.60	America	Plasma	LC-MS	PLS-DA (VIP ≥ 2)
	NPDR patients (*n* = 49)	59.4 ± 11.3	61.40				
Haines (2018)	PDR (*n* = 9)	41 ± 10		America	Vitreous	UHPLC-MS	ANOVA, *t*-test (*p* < 0.05)
	rhegmatogenous RD (*n* = 25), and controls (*n* = 8)	68 ± 6 (controls); 62 ± 10 (rhegmatogenous RD)					
Zhu (2019)	PDR (*n* = 21)	49 (46–56.5)	42.86	China	Plasma	UPLC Q-TOF-MS	*t*-test (*p* < 10^−5^), AUC ≥ 0.95 & PLS-DA (VIP > 1)
	NDR (duration ≥ 10y) (*n* = 21)	55 (50–58)	42.86				
Wang (2019)	PDR (*n* = 28) (Vitreous); PDR (*n* = 23) (AH)	49.61 (26–65)	42.86	China	Vitreous & AH	GC-TOF-MS	OPLS-DA (VIP > 1), Mann-Whitney U test (*p* < 0.05)
	non-diabetic patients with MH (*n* = 22) (Vitreous); non-diabetic patients with cataract (*n* = 25) (AH)	53.95 (32–71)	36.36				
Paris (2015)	PDR (*n* = 20)			Tokyo	Vitreous	HILIC & RPLC QTOF-MS	Welch’s *t* test (*p* < 0.01, FC > 2)
	controls (*n* = 31)						
Barba (2010)	PDR (*n* = 22)			Spain	Vitreous	NMR	
	non-diabetic patients with MH (*n* = 22)						
ABHARY (2009)	no diabetic retinopathy (*n* = 330)			Australia	Serum	LC-MS	Hierarchical multiple regression (*p* < 0.05)
	PDR (*n* = 101)						
Tomita (2020)	PDR (*n* = 43)	58.1 ± 13.6	77.1	Tokyo	Vitreous	UHPLC-MS	*t*-test (FDR < 0.05)
	controls (*n* = 21)	69.4 ± 7.0	42.1				
Lin (2020)	PDR (*n* = 31)			America	Vitreous	LC/MS/MS	*t*-test (*p* < 0.05)
	controls (*n* = 13)						
Yun (2020)	PDR (*n* = 51)	62.60 (11.60)	60.2	Korea	Serum	LC-MS	Logistic regression analysis
	NPDR (*n* = 123)	61.18 (11.87)	50.29				
Ye (2021)	PDR (*n* = 45)	59.9 ± 11.3	55.56	China	Fecal	UPLC-MS	PLS-DA (VIP > 1), *p* < 0.05, |log2(FC)| > 1
	diabetic patients without DR (*n* = 90)	60.9 ± 9.9	55.56				
Wang (2022)	PDR (*n* = 88) (Plasma); PDR (*n* = 51) (Vitreous)	55.3 ± 9.7 (Plasma); 54.9 ± 9.2 (Vitreous)	51.80	China	Plasma & Vitreous	UPLC-MS/MS	FDR < 0.05, OPLS-DA (VIP > 1.0), FC > 1.2 or <0.83 and multivariate analysis
	nondiabetic controls (*n* = 51) (Plasma); nondiabetic controls(*n* = 23) (Vitreous)	67.0 ± 8.1 (Plasma); 67.1 ± 8.8 (Vitreous)	36.49				

ROP = retinopathy of prematurity; nAMD = Neovascular age-related macular degeneration; PCV = polypoidal choroidal vasculopathy; PDR = proliferative diabetic retinopathy; NPDR = non-proliferative diabetic retinopathy; RD = retinal detachment; MH = macular hole; GA = gestational age; AH = aqueous humor; LC = Liquid Chromatogram; MS = mass spectrometry; UPLC = Ultra Performance Liquid Chromatography; GC = gas chromatography; TOF = time of flight; Q-TOF = Quadrupole-Time of Flight; OPLS-DA = orthogonal projections to latent structures discriminant analysis; PLS-DA = partial least squares discriminant analysis; ANOVA = one-way analysis of variance; FC = fold change; VIP = variable importance in projection; FDR = false discovery rate.

**Table 2:** Results of the Pathway Analysis of Metabolic Biomarkers.

Pathway Name	Match	*p*	FDR	Impact	Match Details
Plasma					
Arginine biosynthesis	7/14	<0.0001	0.0003	0.6244	L-Glutamate; L-Arginine; L-Citrulline; L-Aspartate; Carbamoyl phosphate; L-Glutamine; Fumarate
Aminoacyl-tRNA biosynthesis	11/48	<0.0001	0.0014	<0.0001	L-Phenylalanine; L-Arginine; L-Glutamine; L-Aspartate; L-Methionine; L-Valine; L-Lysine; L-Tryptophan; L-Tyrosine; L-Proline; L-Glutamate
AH					
Aminoacyl-tRNA biosynthesis	10/48	<0.0001	0.0001	0.1667	L-Phenylalanine; L-Glutamine; L-Serine; L-Methionine; L-Lysine; L-Leucine; L-Tryptophan; L-Tyrosine; L-Proline; L-Glutamate
Glyoxylate and dicarboxylate metabolism	6/32	0.0003	0.0118	0.1455	cis-Aconitate; L-Serine; L-Glutamate; D-Glycerate; Isocitrate; L-Glutamine
Vitreous					
Aminoacyl-tRNA biosynthesis	9/48	<0.0001	0.0005	<0.0001	L-Phenylalanine; L-Glutamine; Glycine; L-Valine; L-Alanine; L-Lysine; L-Leucine; L-Threonine; L-Proline
Alanine, aspartate and glutamate metabolism	7/28	<0.0001	0.0005	0.2484	N-Acetyl-L-aspartate; L-Alanine; L-Glutamine; 2-Oxoglutaramate; Pyruvate; Succinate; 2-Oxoglutarate
Arginine biosynthesis	5/14	<0.0001	0.0009	0.2893	L-Citrulline; L-Ornithine; L-Glutamine; 2-Oxoglutarate; N-Acetyl-L-glutamate
Glycine, serine and threonine metabolism	7/33	<0.0001	0.0009	0.3426	N, N-Dimethylglycine; L-Cystathionine; Glycine; L-Threonine; D-Glycerate; Creatine; Pyruvate
Valine, leucine and isoleucine biosynthesis	3/8	0.0014	0.0235	<0.0001	L-Threonine; L-Leucine; L-Valine
Glyoxylate and dicarboxylate metabolism	5/32	0.0025	0.0346	0.1852	Glycine; D-Glycerate; Acetate; Pyruvate; L-Glutamine

AH = aqueous humor; FDR = false discovery rate.

**Table 3:** Classification/Prediction Potential of Biomarker Panels.

References	Discriminant Models	Discriminant Group; Precision
ROP		
Yang (2020)	Altered metabolites	All AUC > 0.5; C3DC (AUC = 0.914, sen = 97.5%, and spe = 68.3%); glycine (AUC = 0.659, sen = 92.5%, and spe = 58.5%)
Zhou (2021)	Altered metabolites	The AUC values for citrulline, creatinine, arginine, and aminoadipic acid were 0.7221, 0.7000, 0.6759, and 0.6545; The combination of the 4 altered metabolites (AUC = 0.8703)
Zhou (2020)	Altered metabolites	10 metabolites obtained AUC larger than 0.7 under positive ion mode; Under negative ion mode, 5 metabolites obtained AUC larger than 0.7.
AMD		
Mitchell (2018)	159 differential features	Accuracy = 96.1% (training set); 75.6% (test set)
Li (2016)	differential features	LPA (18:2), LPC (20:4), PC (20:1p/19:1), SM (d16:0/22:2), PAF (35:4), PC (16:0/22:5) and PC (18:1/20:4) are evaluated separately, AUC is greater than or equal 0.8
Liu (2020)	Demographic characteristics and panel metabolites(hypoxanthine, L-2-amino-3-(1-pyrazolyl)propanoic acid, linoleic acid, maleic acid, ribonolactone) (PM vs. control);	AUC = 0.906; sen = 0.877; spe = 0.684 (PM vs. control);
	Demographic characteristics and panel metabolites(5-hydroxylysine, caproic acid, D-tagatose, glyceraldehyde, hydroxyphenyllactic acid, L-2-amino-3-(1-pyrazolyl)propanoic acid, linoleic acid, pipecolic acid, pyruvic acid, and ribonolactone) (AMD vs. control);	AUC = 0.971; sen = 0.963; spe = 0.907 (AMD vs. control);
	Demographic characteristics and panel metabolites(hypoxanthine,L-2-Amino-3-(1-pyrazolyl)propanoicacid,linoleic acid, maleic acid, pipecolic acid, pyruvic acid, ribonolactone, 5-hydroxydopamine, and phenylpyruvic acid) (PCV vs. control);	AUC = 0.948; sen = 0.901; spe = 0.853 (PCV vs. control);
PDR		
Sumarriva (2019)	219 differential features	Accuracy = 91.7%
Zhu (2019)	Fumaric acid, uridine, acetic acid, and cytidine	AUCs = 0.96, 0.95, 1.0, and 0.95, respectively
Wang (2019)	AH: D-2,3-dihydroxypropanoic acid, isocitric acid, fructose 6-phosphate, and L-lactic acid	(AH)AUC = 0.965, sen = 88%, spe = 95.7%
	Vitreous: pyroglutamic acid and pyruvic acid	(Vitreous) AUC = 0.965, sen = 88%, spe = 95.7%

AMD = age-related macular degeneration; PCV = polypoidal choroidal vasculopathy; ROP = retinopathy of prematurity; PDR = proliferative diabetic retinopathy; sen = sensitivity; spe = specificity; AUC = area under curve.

**Table 4:** Performance of Different Classifiers.

	Accuracy	Sensitivity	Specificity	Precision	F1-Score	AUC (Test)
neg-Han (2020)						
Logistic-all	0.643	0.500	0.750	0.600	0.545	0.578
Logistic-step	0.786	0.625	1.000	1.000	0.769	0.833
RF	0.929	1.000	0.800	0.900	0.947	0.900
SVM	0.786	0.800	0.750	0.889	0.842	0.744
XGBoost	0.714	0.889	0.400	0.727	0.800	0.644
pos-Han (2020)						
Logistic-all	0.714	0.750	0.800	0.600	0.667	0.700
Logistic-step	0.929	1.000	0.900	0.800	0.889	0.900
RF	0.929	1.000	0.800	0.900	0.947	0.900
SVM	0.857	0.818	1.000	1.000	0.900	0.800
XGBoost	0.929	1.000	0.800	0.900	0.947	0.900
AH-Wang (2019)						
Logistic-all	0.733	0.667	0.833	0.857	0.750	0.652
Logistic-step	0.600	0.556	0.667	0.714	0.625	0.643
RF	0.867	0.875	0.857	0.857	0.857	0.866
SVM	0.800	0.857	0.750	0.750	0.800	0.804
XGBoost	0.733	0.625	0.857	0.833	0.714	0.741
Vit-Wang (2019)						
Logistic-all	0.556	0.600	0.500	0.600	0.600	0.525
Logistic-step	0.722	0.778	0.667	0.700	0.737	0.725
RF	0.833	0.750	0.900	0.857	0.800	0.825
SVM	0.778	0.750	0.800	0.750	0.750	0.775
XGBoost	0.778	0.750	0.800	0.750	0.750	0.775
Yun (2020)						
Logistic-all	0.811	0.909	0.333	0.870	0.889	0.478
Logistic-step	0.792	0.889	0.250	0.870	0.879	0.472
RF	0.792	0.429	0.848	0.300	0.353	0.638
SVM	0.868	0.500	0.898	0.286	0.364	0.621
XGBoost	0.792	0.571	0.826	0.333	0.421	0.699
Barca (2020)						
Logistic-all	0.667	0.600	0.778	0.818	0.692	0.671
Logistic-step	0.750	0.692	0.818	0.818	0.750	0.748
RF	0.667	0.538	0.818	0.778	0.636	0.678
SVM	0.667	0.727	0.615	0.615	0.667	0.671
XGBoost	0.792	0.692	0.909	0.900	0.783	0.801

## References

[1] Hirschi K., Goodell M. (2001). Common origins of blood and blood vessels in adults?. Differentiation.

[2] Afzal A., Shaw L.C., Ljubimov A.V., Ljubimov A.V., Boulton M.E., Segal M.S., Grant M.B. (2007). Retinal and choroidal microangiopathies: Therapeutic opportunities. Microvasc. Res..

[3] Carmeliet P., Jain R.K. (2000). Angiogenesis in cancer and other diseases. Nature.

[4] Yan W., Peng Y.R., van Zyl T., Regev A., Shekhar K., Juric D., Sanes J.R. (2020). Cell Atlas of The Human Fovea and Peripheral Retina. Sci. Rep..

[5] Al-Latayfeh M., Silva P.S., Sun J.K., Aiello L.P. (2012). Antiangiogenic therapy for ischemic retinopathies. Cold Spring Harb. Perspect. Med..

[6] Chheda L.V., Ferketich A.K., Carroll C.P., Moyer P.D., Kurz D.E., Kurz P.A. (2012). Smoking as a risk factor for choroidal neovascularization secondary to presumed ocular histoplasmosis syndrome. Ophthalmology.

[7] Yanai R., Chen S., Uchi S.H., Nanri T., Connor K.M., Kimura K. (2018). Attenuation of choroidal neovascularization by dietary intake of ω-3 long-chain polyunsaturated fatty acids and lutein in mice. PloS ONE.

[8] Tan J.S., Wang J.J., Flood V., Rochtchina E., Smith W., Mitchell P. (2008). Dietary antioxidants and the long-term incidence of age-related macular degeneration: The Blue Mountains Eye Study. Ophthalmology.

[9] Xu X.D., Li K.R., Li X.M., Yao J., Qin J., Yan B. (2014). Long non-coding RNAs: New players in ocular neovascularization. Mol. Biol. Rep..

[10] Khan S.R., Manialawy Y., Wheeler M.B., Cox B.J. (2019). Unbiased data analytic strategies to improve biomarker discovery in precision medicine. Drug Discov. Today.

[11] Yazdani M., Elgstøen K.B.P., Rootwelt H., Shahdadfar A., Utheim Ø.A., Utheim T.P. (2019). Tear Metabolomics in Dry Eye Disease: A Review. Int. J. Mol. Sci..

[12] Holt-Lunstad J. (2021). Loneliness and Social Isolation as Risk Factors: The Power of Social Connection in Prevention. Am. J. Lifestyle Med..

[13] Chen L., Gao Y., Wang L.Z., Cheung N., Tan G.S.W., Cheung G.C.M., Beuerman R.W., Wong T.Y., Chan E.C.Y., Zhou L. (2018). Recent advances in the applications of metabolomics in eye research. Anal. Chim. Acta.

[14] Cunha-Vaz J. (1979). The blood-ocular barriers. Surv. Ophthalmol..

[15] Saint-Geniez M., D’Amore P.A. (2004). Development and pathology of the hyaloid, choroidal and retinal vasculature. Int. J. Dev. Biol..

[16] Hou X.W., Wang Y., Pan C.W. (2020). Metabolomics in Age-Related Macular Degeneration: A Systematic Review. Investig. Ophthalmol. Vis. Sci..

[17] Campochiaro P.A. (2015). Molecular pathogenesis of retinal and choroidal vascular diseases. Prog. Retin. Eye Res..

[18] Nilsson A.K., Andersson M.X., Sjöbom U., Hellgren G., Lundgren P., Pivodic A., Smith L.E.H., Hellström A. (2021). Sphingolipidomics of serum in extremely preterm infants: Association between low sphingosine-1-phosphate levels and severe retinopathy of prematurity. Biochim. Biophys. Acta Mol. Cell Biol. Lipids.

[19] Ye P., Zhang X., Xu Y., Xu J., Song X., Yao K. (2021). Alterations of the Gut Microbiome and Metabolome in Patients With Proliferative Diabetic Retinopathy. Front. Microbiol..

[20] Li M., Zhang X., Liao N., Ye B., Peng Y., Ji Y., Wen F. (2016). Analysis of the Serum Lipid Profile in Polypoidal Choroidal Vasculopathy. Sci. Rep..

[21] Wang H., Fang J., Chen F., Sun Q., Xu X., Lin S.H., Liu K. (2020). Metabolomic profile of diabetic retinopathy: A GC-TOFMS-based approach using vitreous and aqueous humor. Acta Diabetol..

[22] Han G., Wei P., He M., Teng H., Chu Y. (2020). Metabolomic Profiling of the Aqueous Humor in Patients with Wet Age-Related Macular Degeneration Using UHPLC-MS/MS. J. Proteome Res..

[23] Wang H., Li S., Wang C., Wang Y., Fang J., Liu K. (2022). Plasma and Vitreous Metabolomics Profiling of Proliferative Diabetic Retinopathy. Investig. Ophthalmol. Vis. Sci..

[24] Zhou Y., Xu Y., Zhang X., Huang Q., Tan W., Yang Y., He X., Yoshida S., Zhao P., Li Y. (2021). Plasma levels of amino acids and derivatives in retinopathy of prematurity. Int. J. Med. Sci..

[25] Zhou Y., Xu Y., Zhang X., Zhao P., Gong X., He M., Cao J., Jiang B., Yoshida S., Li Y. (2020). Plasma metabolites in treatment-requiring retinopathy of prematurity: Potential biomarkers identified by metabolomics. Exp. Eye Res..

[26] Zhu X.R., Yang F.Y., Lu J., Zhang H.R., Sun R., Zhou J.B., Yang J.K. (2019). Plasma metabolomic profiling of proliferative diabetic retinopathy. Nutr. Metab..

[27] Luo D., Deng T., Yuan W., Deng H., Jin M. (2017). Plasma metabolomic study in Chinese patients with wet age-related macular degeneration. BMC Ophthalmol..

[28] Liu K., Fang J., Jin J., Zhu S., Xu X., Xu Y., Ye B., Lin S.H., Xu X. (2020). Serum Metabolomics Reveals Personalized Metabolic Patterns for Macular Neovascular Disease Patient Stratification. J. Proteome Res..

[29] Deng Y., Shuai P., Wang H., Zhang S., Li J., Du M., Huang P., Qu C., Huang L. (2021). Untargeted metabolomics for uncovering plasma biological markers of wet age-related macular degeneration. Aging.

[30] Yang Y., Wu Z., Li S., Yang M., Xiao X., Lian C., Wen W., He H., Zeng J., Wang J. (2020). Targeted Blood Metabolomic Study on Retinopathy of Prematurity. Investig. Ophthalmol. Vis. Sci..

[31] Haines N.R., Manoharan N., Olson J.L., D’Alessandro A., Reisz J.A. (2018). Metabolomics Analysis of Human Vitreous in Diabetic Retinopathy and Rhegmatogenous Retinal Detachment. J. Proteome Res..

[32] Mitchell S.L., Uppal K., Williamson S.M., Liu K., Burgess L.G., Tran V., Umfress A.C., Jarrell K.L., Cooke Bailey J.N., Agarwal A. (2018). The Carnitine Shuttle Pathway is Altered in Patients With Neovascular Age-Related Macular Degeneration. Investig. Ophthalmol. Vis. Sci..

[33] Sumarriva K., Uppal K., Ma C., Herren D.J., Wang Y., Chocron I.M., Warden C., Mitchell S.L., Burgess L.G., Goodale M.P. (2019). Arginine and Carnitine Metabolites Are Altered in Diabetic Retinopathy. Investig. Ophthalmol. Vis. Sci..

[34] Lin A.L., Roman R.J., Regan K.A., Bolch C.A., Chen C.J., Iyer S.S.R. (2020). Eicosanoid Profiles in the Vitreous Humor of Patients with Proliferative Diabetic Retinopathy. Int. J. Mol. Sci..

[35] Paris L.P., Johnson C.H., Aguilar E., Usui Y., Cho K., Hoang L.T., Feitelberg D., Benton H.P., Westenskow P.D., Kurihara T. (2016). Global metabolomics reveals metabolic dysregulation in ischemic retinopathy. Metab. Off. J. Metab. Soc..

[36] Tomita Y., Cagnone G., Fu Z., Cakir B., Kotoda Y., Asakage M., Wakabayashi Y., Hellström A., Joyal J.S., Talukdar S. (2021). Vitreous metabolomics profiling of proliferative diabetic retinopathy. Diabetologia.

[37] Barba I., Garcia-Ramírez M., Hernández C., Alonso M.A., Masmiquel L., García-Dorado D., Simó R. (2010). Metabolic fingerprints of proliferative diabetic retinopathy: An 1H-NMR-based metabonomic approach using vitreous humor. Investig. Ophthalmol. Vis. Sci..

[38] Chao de la Barca J.M., Rondet-Courbis B., Ferré M., Muller J., Buisset A., Leruez S., Plubeau G., Macé T., Moureauzeau L., Chupin S. (2020). A Plasma Metabolomic Profiling of Exudative Age-Related Macular Degeneration Showing Carnosine and Mitochondrial Deficiencies. J. Clin. Med..

[39] Lambert V., Hansen S., Schoumacher M., Lecomte J., Leenders J., Hubert P., Herfs M., Blacher S., Carnet O., Yip C. (2020). Pyruvate dehydrogenase kinase/lactate axis: A therapeutic target for neovascular age-related macular degeneration identified by metabolomics. J. Mol. Med..

[40] Osborn M.P., Park Y., Parks M.B., Burgess L.G., Uppal K., Lee K., Jones D.P., Brantley M.A. (2013). Metabolome-wide association study of neovascular age-related macular degeneration. PloS ONE.

[41] Ozcan Y., Huseyin G., Sonmez K. (2020). Evaluation of Plasma Amino Acid Levels in Preterm Infants and Their Potential Correlation with Retinopathy of Prematurity. J. Ophthalmol..

[42] Yun J.H., Kim J.M., Jeon H.J., Oh T., Choi H.J., Kim B.J. (2020). Metabolomics profiles associated with diabetic retinopathy in type 2 diabetes patients. PloS ONE.

[43] Abhary S., Kasmeridis N., Burdon K.P., Kuot A., Whiting M.J., Yew W.P., Petrovsky N., Craig J.E. (2009). Diabetic retinopathy is associated with elevated serum asymmetric and symmetric dimethylarginines. Diabetes Care.

[44] Lee L.C., Liong C.Y., Jemain A.A. (2018). Partial least squares-discriminant analysis (PLS-DA) for classification of high-dimensional (HD) data: A review of contemporary practice strategies and knowledge gaps. Analyst.

[45] Einav S., O’Connor M. (2019). *p*-values and significance: The null hypothesis that they are not related is correct. J. Crit. Care.

[46] Sun Y.V., Hu Y.J. (2016). Integrative Analysis of Multi-omics Data for Discovery and Functional Studies of Complex Human Diseases. Adv. Genet..

[47] Wan-Ibrahim W.I., Singh V.A., Hashim O.H., Abdul-Rahman P.S. (2016). Biomarkers for Bone Tumors: Discovery from Genomics and Proteomics Studies and Their Challenges. Mol. Med..

[48] Vavricka C.J., Hasunuma T., Kondo A. (2020). Dynamic Metabolomics for Engineering Biology: Accelerating Learning Cycles for Bioproduction. Trends Biotechnol..

[49] Palli R., Palshikar M.G., Thakar J. (2019). Executable pathway analysis using ensemble discrete-state modeling for large-scale data. PLoS Comput. Biol..

[50] Pang Z., Chong J., Zhou G., de Lima Morais D.A., Chang L., Barrette M., Gauthier C., Jacques P., Li S., Xia J. (2021). MetaboAnalyst 5.0: Narrowing the gap between raw spectra and functional insights. Nucleic Acids Res..

[51] Sorigue M., Cañamero E., Sancho J.M. (2020). Precision medicine in follicular lymphoma: Focus on predictive biomarkers. Hematol. Oncol..

[52] Gevaert O., De Smet F., Timmerman D., Moreau Y., De Moor B. (2006). Predicting the prognosis of breast cancer by integrating clinical and microarray data with Bayesian networks. Bioinformatics.

[53] Treps L., Conradi L.C., Harjes U., Carmeliet P. (2016). Manipulating Angiogenesis by Targeting Endothelial Metabolism: Hitting the Engine Rather than the Drivers-A New Perspective?. Pharmacol. Rev..

[54] Morris S.M. (2009). Recent advances in arginine metabolism: Roles and regulation of the arginases. Br. J. Pharmacol..

[55] Cantelmo A.R., Conradi L.C., Brajic A., Goveia J., Kalucka J., Pircher A., Chaturvedi P., Hol J., Thienpont B., Teuwen L.A. (2016). Inhibition of the Glycolytic Activator PFKFB3 in Endothelium Induces Tumor Vessel Normalization, Impairs Metastasis, and Improves Chemotherapy. Cancer Cell.

[56] Lieth E., Barber A.J., Xu B., Dice C., Ratz M.J., Tanase D., Strother J.M. (1998). Glial reactivity and impaired glutamate metabolism in short-term experimental diabetic retinopathy. Penn State Retina Research Group. Diabetes.

[57] Oh H.S., Oh S.K., Lee J.S., Wu C., Lee S.J. (2017). Effects of l-arginine on growth hormone and insulin-like growth factor 1. Food Sci. Biotechnol..

[58] Hellström A., Engström E., Hård A.L., Albertsson-Wikland K., Carlsson B., Niklasson A., Löfqvist C., Svensson E., Holm S., Ewald U. (2003). Postnatal serum insulin-like growth factor I deficiency is associated with retinopathy of prematurity and other complications of premature birth. Pediatrics.

[59] Dulak J., Józkowicz A., Dembinska-Kiec A., Guevara I., Zdzienicka A., Zmudzinska-Grochot D., Florek I., Wójtowicz A., Szuba A., Cooke J.P. (2000). Nitric oxide induces the synthesis of vascular endothelial growth factor by rat vascular smooth muscle cells. Arterioscler. Thromb. Vasc. Biol..

[60] Thibeault S., Rautureau Y., Oubaha M., Faubert D., Wilkes B.C., Delisle C., Gratton J.P. (2010). S-nitrosylation of beta-catenin by eNOS-derived NO promotes VEGF-induced endothelial cell permeability. Mol. Cell.

[61] Smith T.L., Oubaha M., Cagnone G., Boscher C., Kim J.S., El Bakkouri Y., Zhang Y., Chidiac R., Corriveau J., Delisle C. (2021). eNOS controls angiogenic sprouting and retinal neovascularization through the regulation of endothelial cell polarity. Cell. Mol. Life Sci. CMLS.

[62] Palmer R.M., Ashton D.S., Moncada S. (1988). Vascular endothelial cells synthesize nitric oxide from L-arginine. Nature.

[63] Horowitz S., Binion D.G., Nelson V.M., Kanaa Y., Javadi P., Lazarova Z., Andrekopoulos C., Kalyanaraman B., Otterson M.F., Rafiee P. (2007). Increased arginase activity and endothelial dysfunction in human inflammatory bowel disease. Am. J. Physiol. Gastrointest. Liver Physiol..

[64] Leal J., Teixeira-Santos L., Pinho D., Afonso J., Carvalho J., de Lourdes Bastos M., Albino-Teixeira A., Fraga S., Sousa T. (2019). L-proline supplementation improves nitric oxide bioavailability and counteracts the blood pressure rise induced by angiotensin II in rats. Nitric Oxide Biol. Chem..

[65] Lenis Y.Y., Elmetwally M.A., Maldonado-Estrada J.G., Bazer F.W. (2017). Physiological importance of polyamines. Zygote.

[66] Ola M.S., Alhomida A.S., LaNoue K.F. (2019). Gabapentin Attenuates Oxidative Stress and Apoptosis in the Diabetic Rat Retina. Neurotox. Res..

[67] Hung C.M., Garcia-Haro L., Sparks C.A., Guertin D.A. (2012). mTOR-dependent cell survival mechanisms. Cold Spring Harb. Perspect. Biol..

[68] Wei J., Jiang H., Gao H., Wang G. (2016). Blocking Mammalian Target of Rapamycin (mTOR) Attenuates HIF-1α Pathways Engaged-Vascular Endothelial Growth Factor (VEGF) in Diabetic Retinopathy. Cell. Physiol. Biochem. Int. J. Exp. Cell. Physiol. Biochem. Pharmacol..

[69] Jacot J.L., Sherris D. (2011). Potential Therapeutic Roles for Inhibition of the PI3K/Akt/mTOR Pathway in the Pathophysiology of Diabetic Retinopathy. J. Ophthalmol..

[70] Colak E., Majkic-Singh N., Zoric L., Radosavljevic A., Kosanovic-Jakovic N. (2012). The role of CRP and inflammation in the pathogenesis of age-related macular degeneration. Biochem. Med..

[71] Liu X., Zhao P., Tang S., Lu F., Hu J., Lei C., Yang X., Lin Y., Ma S., Yang J. (2010). Association study of complement factor H, C2, CFB, and C3 and age-related macular degeneration in a Han Chinese population. Retina.

[72] Lyzogubov V.V., Tytarenko R.G., Jha P., Liu J., Bora N.S., Bora P.S. (2010). Role of ocular complement factor H in a murine model of choroidal neovascularization. Am. J. Pathol..

[73] McHarg S., Clark S.J., Day A.J., Bishop P.N. (2015). Age-related macular degeneration and the role of the complement system. Mol. Immunol..

[74] Nakajima T., Nakajima E., Shearer T.R., Azuma M. (2013). Concerted inhibition of HIF-1α and -2α expression markedly suppresses angiogenesis in cultured RPE cells. Mol. Cell. Biochem..

[75] Ryan H.E., Lo J., Johnson R.S. (1998). HIF-1 alpha is required for solid tumor formation and embryonic vascularization. EMBO J..

[76] Bhattarai D., Xu X., Lee K. (2018). Hypoxia-inducible factor-1 (HIF-1) inhibitors from the last decade (2007 to 2016): A "structure-activity relationship" perspective. Med. Res. Rev..

[77] Dong A., Seidel C., Snell D., Ekawardhani S., Ahlskog J.K., Baumann M., Shen J., Iwase T., Tian J., Stevens R. (2014). Antagonism of PDGF-BB suppresses subretinal neovascularization and enhances the effects of blocking VEGF-A. Angiogenesis.

[78] Jaffe G.J., Ciulla T.A., Ciardella A.P., Devin F., Dugel P.U., Eandi C.M., Masonson H., Monés J., Pearlman J.A., Quaranta-El Maftouhi M. (2017). Dual Antagonism of PDGF and VEGF in Neovascular Age-Related Macular Degeneration: A Phase IIb, Multicenter, Randomized Controlled Trial. Ophthalmology.

[79] Datta S., Cano M., Ebrahimi K., Wang L., Handa J.T. (2017). The impact of oxidative stress and inflammation on RPE degeneration in non-neovascular AMD. Prog. Retin. Eye Res..

[80] Yam M., Engel A.L., Wang Y., Zhu S., Hauer A., Zhang R., Lohner D., Huang J., Dinterman M., Zhao C. (2019). Proline mediates metabolic communication between retinal pigment epithelial cells and the retina. J. Biol. Chem..

[81] Thurman J.M., Renner B., Kunchithapautham K., Ferreira V.P., Pangburn M.K., Ablonczy Z., Tomlinson S., Holers V.M., Rohrer B. (2009). Oxidative stress renders retinal pigment epithelial cells susceptible to complement-mediated injury. J. Biol. Chem..

[82] Xu H., Chen M. (2016). Targeting the complement system for the management of retinal inflammatory and degenerative diseases. Eur. J. Pharmacol..

[83] Nozaki M., Raisler B.J., Sakurai E., Sarma J.V., Barnum S.R., Lambris J.D., Chen Y., Zhang K., Ambati B.K., Baffi J.Z. (2006). Drusen complement components C3a and C5a promote choroidal neovascularization. Proc. Natl. Acad. Sci. USA.

[84] Ambati J., Atkinson J.P., Gelfand B.D. (2013). Immunology of age-related macular degeneration. Nat. Rev. Immunol..

[85] Rohrer B., Long Q., Coughlin B., Renner B., Huang Y., Kunchithapautham K., Ferreira V.P., Pangburn M.K., Gilkeson G.S., Thurman J.M. (2010). A targeted inhibitor of the complement alternative pathway reduces RPE injury and angiogenesis in models of age-related macular degeneration. Adv. Exp. Med. Biol..

[86] Polet F., Feron O. (2013). Endothelial cell metabolism and tumour angiogenesis: Glucose and glutamine as essential fuels and lactate as the driving force. J. Intern. Med..

[87] Wert K.J., Velez G., Kanchustambham V.L., Shankar V., Evans L.P., Sengillo J.D., Zare R.N., Bassuk A.G., Tsang S.H., Mahajan V.B. (2020). Metabolite therapy guided by liquid biopsy proteomics delays retinal neurodegeneration. EBioMedicine.

[88] Kanow M.A., Giarmarco M.M., Jankowski C.S., Tsantilas K., Engel A.L., Du J., Linton J.D., Farnsworth C.C., Sloat S.R., Rountree A. (2017). Biochemical adaptations of the retina and retinal pigment epithelium support a metabolic ecosystem in the vertebrate eye. eLife.

[89] De Bock K., Georgiadou M., Schoors S., Kuchnio A., Wong B.W., Cantelmo A.R., Quaegebeur A., Ghesquière B., Cauwenberghs S., Eelen G. (2013). Role of PFKFB3-driven glycolysis in vessel sprouting. Cell.

[90] Xu Y., An X., Guo X., Habtetsion T.G., Wang Y., Xu X., Kandala S., Li Q., Li H., Zhang C. (2014). Endothelial PFKFB3 plays a critical role in angiogenesis. Arterioscler. Thromb. Vasc. Biol..

[91] Liu Z., Yan S., Wang J., Xu Y., Wang Y., Zhang S., Xu X., Yang Q., Zeng X., Zhou Y. (2017). Endothelial adenosine A2a receptor-mediated glycolysis is essential for pathological retinal angiogenesis. Nat. Commun..

[92] Al-Shabrawey M., Mussell R., Kahook K., Tawfik A., Eladl M., Sarthy V., Nussbaum J., El-Marakby A., Park S.Y., Gurel Z. (2011). Increased expression and activity of 12-lipoxygenase in oxygen-induced ischemic retinopathy and proliferative diabetic retinopathy: Implications in retinal neovascularization. Diabetes.

[93] Chen L., Ackerman R., Guo A.M. (2012). 20-HETE in neovascularization. Prostaglandins Other Lipid Mediat..

[94] Shao Z., Fu Z., Stahl A., Joyal J.S., Hatton C., Juan A., Hurst C., Evans L., Cui Z., Pei D. (2014). Cytochrome P450 2C8 ω3-long-chain polyunsaturated fatty acid metabolites increase mouse retinal pathologic neovascularization—Brief report. Arterioscler. Thromb. Vasc. Biol..

[95] SanGiovanni J.P., Chew E.Y., Clemons T.E., Davis M.D., Ferris F.L., Gensler G.R., Kurinij N., Lindblad A.S., Milton R.C., Seddon J.M. (2007). The relationship of dietary lipid intake and age-related macular degeneration in a case-control study: AREDS Report No. 20. Arch. Ophthalmol..

[96] Sapieha P., Chen J., Stahl A., Seaward M.R., Favazza T.L., Juan A.M., Hatton C.J., Joyal J.S., Krah N.M., Dennison R.J. (2012). Omega-3 polyunsaturated fatty acids preserve retinal function in type 2 diabetic mice. Nutr. Diabetes.

[97] Connor K.M., SanGiovanni J.P., Lofqvist C., Aderman C.M., Chen J., Higuchi A., Hong S., Pravda E.A., Majchrzak S., Carper D. (2007). Increased dietary intake of omega-3-polyunsaturated fatty acids reduces pathological retinal angiogenesis. Nat. Med..

[98] Lains I., Mendez K.M., Gil J.Q., Miller J.B., Kelly R.S., Barreto P., Kim I.K., Vavvas D.G., Murta J.N., Liang L. (2022). Urinary Mass Spectrometry Profiles in Age-Related Macular Degeneration. J. Clin. Med..

